# Synthesis and Biological Activity of Diastereomeric and Geometric Analogs of Calcipotriol, PRI-2202 and PRI-2205, Against Human HL-60 Leukemia and MCF-7 Breast Cancer Cells

**DOI:** 10.3390/cancers5041355

**Published:** 2013-10-31

**Authors:** Magdalena Milczarek, Michał Chodyński, Beata Filip-Psurska, Agnieszka Martowicz, Małgorzata Krupa, Krzysztof Krajewski, Andrzej Kutner, Joanna Wietrzyk

**Affiliations:** 1 Ludwik Hirszfeld Institute of Immunology and Experimental Therapy, 12 Weigla, Wroclaw 53-114, Poland; E-Mails: milczarek@iitd.pan.wroc.pl (M.M.); filip@iitd.pan.wroc.pl (B.F.-P.); martowicz@gmail.com (A.M.); 2 Pharmaceutical Research Institute, 8 Rydygiera, Warsaw 01-793, Poland; E-Mails: m.chodynski@ifarm.eu (M.C.); m.krupa@ifarm.eu (M.K.); k.krajewski@ifarm.eu (K.K.); a.kutner@ifarm.eu (A.K.)

**Keywords:** calcipotriol, vitamin D analogs, convergent synthesis, antiproliferative activity, cancer, combined treatment, VDR

## Abstract

Diastereomeric and geometric analogs of calcipotriol, PRI-2202 and PRI-2205, were synthesized as advanced intermediates from vitamin D C-22 benzothiazoyl sulfones and side-chain aldehydes using our convergent strategy. Calcitriol, calcipotriol (PRI-2201) and tacalcitol (PRI-2191) were used as the reference compounds. Among a series of tested analogs the diastereomeric analog PRI-2202 showed the strongest antiproliferative activity on the human breast cancer cell line MCF-7, whereas the geometric analog PRI-2205 was the weakest. Both analogs were less potent in antiproliferative activity against HL-60 cells compared to the reference compounds. The ability to potentiate antiproliferative effect of cisplatin or doxorubicin against HL-60 cells or that of tamoxifen against the MCF-7 cell line was observed at higher doses of PRI-2202 or PRI-2205 than those of the reference compounds. The proapoptotic activity of tamoxifen, expressed as the diminished mitochondrial membrane potential, as well as the increased phosphatidylserine expression, was partially attenuated by calcitriol, PRI-2191, PRI-2201 and PRI-2205. The treatment of the MCF-7 cells with tamoxifen alone resulted in an increase in VDR expression. Moreover, a further increase in VDR expression was observed when the analogs PRI-2201 or PRI-2205, but not PRI-2191, were used in combination with tamoxifen. This observation could partially explain the potentiation of the antiproliferative effect of tamoxifen by vitamin D analogs.

## 1. Introduction

*In vitro* and *in vivo* observations have demonstrated that calcitriol [1,25-dihydroxyvitamin D_3_, 1,25-(OH)_2_D_3_], a hormonally active form of vitamin D_3_, is a potent inhibitor of tumor cell growth. This provides the rationale for using this *seco*-steroid hormone to treat patients with leukemia and various types of cancer [1,2,3,4]. The biological activity of 1,25-(OH)_2_D_3_ is realized mostly via binding to a specific nuclear receptor, the vitamin D receptor (VDR). VDR dimerizes with retinoid X receptor (RXR) and regulates gene expression by acting as a transcription factor which is modulated via ligand binding [5,6,7]. On the other hand, 1,25-(OH)_2_D_3_ can also act independently of the intracellular VDR [8]. Rapid effects of vitamin D, that are not dependent on gene transcription, are realized by binding and activating VDR or membrane-associated steroid binding protein (1,25D_3_-MARRS) [9,10,11]. Although a lot of studies demonstrate the therapeutic potential or protective role of 1,25-(OH)_2_D_3_ the application of potentially effective, hyper-physiological doses of this compound in anticancer treatment is limited by its calcemic activity and subsequent risk of hypercalcemia [2]. These undesired side effects motivated the search for new synthetic analogs with dissociating calcemic and antiproliferative effects.

**Figure 1:**
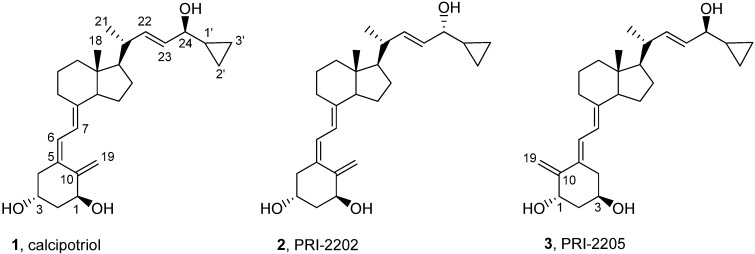
Structures of calcipotriol (**1**, PRI-2201), its C-24 diastereomer (**2**, PRI-2202) and geometric (5*E,7E*) analog (**3**, PRI-2205).

In our previous studies we examined a series of vitamin D_2_ analogs with highly unsaturated and extended side-chains (PRI-1906, PRI-1907, PRI-1908 and PRI-1909) and a series of vitamin D_3_ analogs with hydroxyls at the carbon atom C-24 in the side-chain (PRI-2201, PRI-2202, PRI-2203, PRI-2204 and PRI-2205) for their antiproliferative activity *in vitro* against various human normal and cancer cell lines. We also showed that the vitamin D_3_ metabolite, (24*R*)-1,24-dihydroxyvitamin D_3_ [tacalcitol, (24*R*)-1,24-(OH)_2_D_3_, PRI-2191] revealed higher antitumor and lower calcemic activity as well as lower toxicity than calcitriol [12]. We also observed higher antiproliferative activity of the analog PRI-1906 and its side-chain unsaturated homolog PRI-1907. We showed that the antitumor effect of these analogs could be attributed to the induction of cancer cell differentiation *in vitro* [13,14,15]. Generally, PRI-1906 revealed higher toxicity than PRI-2191, but lower than that of calcitriol and antitumor activity similar to that of PRI-2191 or calcitriol. However, the ability of the PRI-1906 to induce differentiation of mammary adenocarcinoma cells *in vivo* was limited and lower than that of PRI-2191. Moreover, the analog PRI-1907 was significantly more toxic than calcitriol, PRI-1906 and PRI-2191 [16]. The geometric analogs of vitamin D with the reversed (5*E*,7*E*) geometry of the triene system were reported by our [17,18] and other laboratories [19,20] to show the enhanced biological activity, as compared to the natural (5*Z*,7*E*) vitamin D compounds. This is why we designed and synthesized the analog PRI-2205, a geometric analog of calcipotriol (our code PRI-2201) for our further detailed examination. Our interest in diastereomeric analogs resulted in our design and synthesis of the analog PRI-2202 with the reversed chirality at C-24. In our initial study the analogs PRI-2202 and PRI-2205 (Figure 1) have shown effects on cell cycle progression and exerted antiproliferative activity *in vitro* and antitumor activity *in vivo.* Moreover, their toxicity was extremely decreased [18,21]. 

Previously, we described our synthesis of calcipotriol (PRI-2201) from the vitamin D C-22 benzothiazoyl sulfone, having the triene system protected as a convenient Diels Alder SO_2_ adduct [22,23]. This strategy was described in this paper to synthesize the analog PRI-2202 from the same benzothiazoyl sulfone and the analog PRI-2205 from the respective (5*E*,7*E*) sulfone [24].

## 2. Results and Discussion

**Scheme 1:**
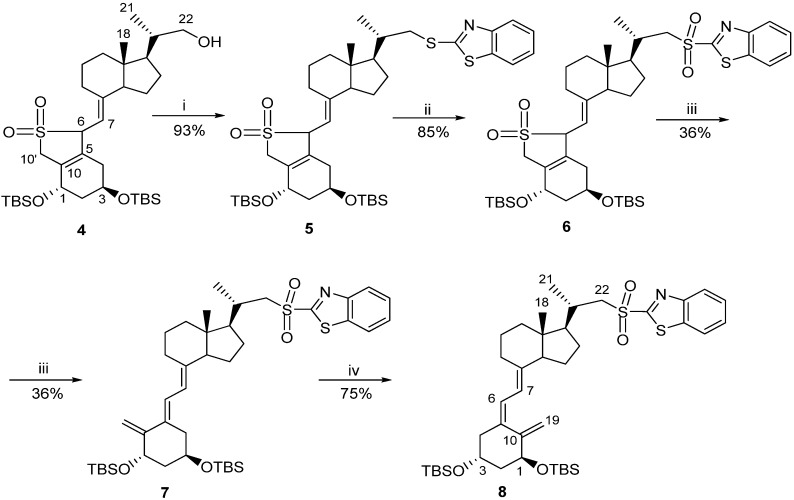
The synthesis of vitamin D advanced intermediates: the (5*E*,7*E*)-benzothiazoyl sulfone **7** and the (5*Z*,7*E*)-benzothiazoyl sulfone **8**.

**Scheme 2:**
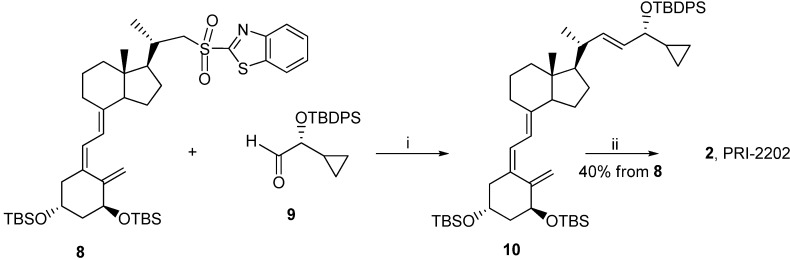
The synthesis of the diastereomeric analog **2** (PRI-2202) from the (5*Z*,7*E*)-benzothiazoyl sulfone **8**.

**Scheme 3:**
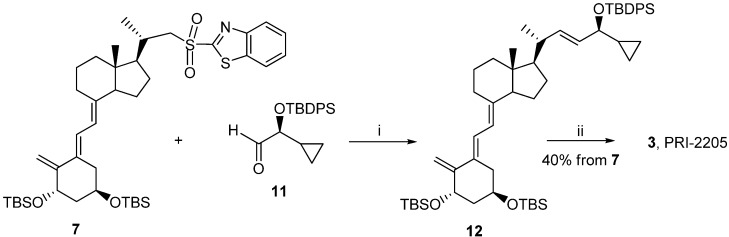
The synthesis of the geometric analog **3** (PRI-2205) from the (5*E*,7*E*)-benzothiazoyl sulfone **7**.

Classic Julia olefination has long been used for the synthesis of the side-chain unsaturated vitamin D analogs. However, in this process a harmful chemical (sodium amalgam) has to be used for the dehydroxy-desulfonylation of the intermediate leading to the final olefin. Later, an improved process of direct olefination was developed using benzothiazoyl sulfone [25]. The coupling of this sulfone with an aldehyde results in the direct olefin formation and the instant release of sulphur dioxide [26]. Our intermediate benzothiazoyl sulfones **7** and **8** (Scheme 1) were obtained from the SO_2_ adduct of C-22 alcohol **4** [22]. This alcohol was prepared by the direct allylic hydroxylation of the (5*E*,7*E*) vitamin D_2_ compound followed by the ozonolytic cleavage of the side-chain of the respective SO_2_ adduct [24]. The sulphide **5** was obtained in high yield from the alcohol **4** using 2-thiobenzothiazole in Mitsunobu conditions. The sulphide **5** was oxidized to the sulfone *6* with molybdenate salt in moderate yield. The deprotection of the sulfone **6** under alkaline conditions gave the (5*E*,7*E*) benzothiazoyl sulfone **7** in a rather low yield that was not improved by extending the reaction time up to three hours under reflux. The photosensitized isomerization of the sulfone **7** in the presence of anthracene [27] gave the sulfone **8** with the natural (5*Z*,7*E*) triene geometry. The analog *2* (PRI-2202) was synthesized (Scheme 2) by the coupling of the benzothiazoyl sulfone **8** with the side-chain aldehyde **9** [23] followed by the deprotection of the intermediate silyl ether **10** with fluoride anion. The analog **3** (Scheme 3, PRI-2205) was obtained from the (5*E*,7*E*) benzothiazoyl sulfone **7** and the side-chain aldehyde **11** [23], as an enantiomer of the aldehyde **9**, using the same process as for the analog **2**. The samples of the analogs PRI-2202 and PRI-2205 for biological evaluation were purified by silica gel chromatography.

*Reagents and conditions*: (i) 2-thiobenzothiazole, triphenylphosphine, DIAD, CH_2_Cl_2_; (ii) ammonium heptamolybdenate hydrate (AHT), H_2_O_2_, EtOH, CH_2_Cl_2_; (iii) NaHCO_3_, EtOH; (iv) toluene-methanol 5:1, anthracene UV.

*Reagents and conditions*: (i) dimethoxyethane (DME), lithium bis(trimethylsilyl)amide; (ii) tetrabutyl-ammonium fluoride solution (1 M in THF), THF.

*Reagents and conditions*: (i) THF, lithium bis (trimethylsilyl) amide; (ii) tetrabutylammonium fluoride solution (1 M in THF), THF.

The activity of the analogs PRI-2202 and PRI-2205 was compared to the reference compounds, calcitriol and calcipotriol (PRI-2201) as well as tacalcitol (PRI-2191). Since the therapeutic efficacy of vitamin D analogs, as single agents, for the systemic treatment of cancer has not yet fulfilled its promise, the alternative concepts are studied to develop cancer treatment strategies that are based on the use of vitamin D compounds in combination with other anticancer therapeutics. 

### 2.1. The Influence of Vitamin D Analogs Combined with Chemotherapeutic Agents on Leukemia and Breast Cancer Cell Line Proliferation

**Figure 2:**
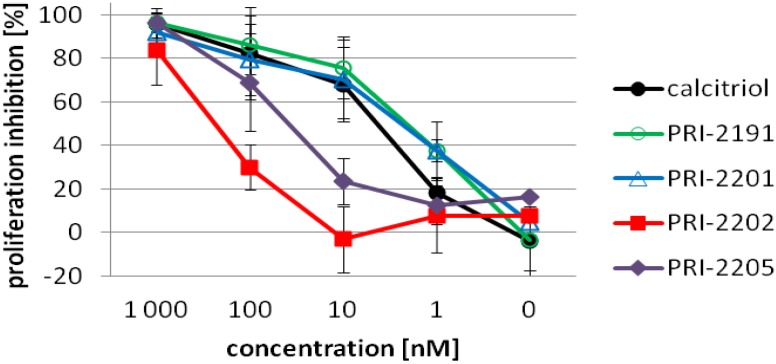
The proliferation inhibition of HL-60 cells by calcitriol or its analogs after 72 h incubation. HL-60 cells were exposed to calcitriol or the PRI-2191, PRI-2201, PRI-2202, PRI-2205 (1000, 100, 10, 1, 0.1 nM) for 72 h.

Figure 2 presents the proliferation profiles of HL-60 cells exposed for 72 h to calcitriol, PRI-2191, PRI-2201, PRI-2202 or PRI-2205. The first three compounds revealed similar strong proliferation inhibition in the range of concentrations between 1,000–10 nM. The activity of the analog PRI-2205 decreased significantly at the concentration of 10 nM. The analog PRI-2202 was less active, revealing high antiproliferative activity only at the highest concentration used. On the basis of these results, the concentrations ranging from 10–0.1 nM for calcitriol, the PRI-2191 and PRI-2201 or 100–1 nM for the PRI-2202 and PRI-2205 were used for further studies.

Table 1 and Table 3 present the proliferation inhibition values after the treatment of HL-60 cells with calcitriol analogs used alone or in combination with doxorubicin (DOX) or cisplatin (CIS). Table 2 and Table 4 present the IC_50_ values (the dose of tested agent which results in 50% inhibition of proliferation of cancer cells) for DOX or CIS used alone or in combination with calcitriol or its analogs. 

When CIS was used alone in the concentration of 10 µg/mL, the proliferation inhibition reaching 98% was observed, giving 100% inhibition in all combined protocols used (data not shown). The most interesting results were observed when calcitriol, PRI-2191 or PRI-2201 were used at the concentration of 1 or 0.1 nM. At these concentrations we could observe the potentiation of the antiproliferative effect of CIS. Statistically significant proliferation inhibition as compared to CIS alone was observed in almost all combinations tested on the HL-60 cell line, following the PRI-2191 pretreatment (Table 1). The tendency to potentiate the antiproliferative activity of CIS was also observed for the PRI-2205, especially in the concentration of 10 nM. 

When analyzing the IC_50_ values for CIS used alone or following the preincubation with vitamin D analogs, we can observe a significant decrease of this value with the increased concentration of vitamin D compounds (Table 2). In the case of 100 nM for calcitriol, the PRI-2191 and PRI-2201 or 10 nM for the PRI-2191 in all concentrations of CIS used, the proliferation was inhibited in above 50% (Table 1).

**Table 1:** Proliferation inhibition of HL-60 cells exposed to calcitriol or its analogs and cisplatin.

Compounds	Vit. D analogs [nM]	Concentration of cisplatin (CIS) [µg/mL]			
		1	0.1	0.01	0
CIS	0	11.2 ± 7.8	1.0 ± 9.4	−2.4 ± 11.2	-
calcitriol	10	65.4 ± 11.2	63.3 ± 7.2	55.3 ± 13.7	64.94 ± 15.2
	1	52.2 ± 24.4	27.3 ± 11.8	21.4 ± 6.6	20.09 ± 7.9
	0.1	35.9 ± 25.5	14.4 ± 13.1	9.0 ± 19.9	20.09 ± 7.9
PRI-2191	10	75.9 ± 14.9 *	76.0 ± 10.5 *	71.7 ± 12.3 *	74.07 ± 13.8
	1	60.8 ± 22.0 *	39.9 ± 15.1 *	40.0 ± 12.8 *	37.8 ± 6.9
	0.1	45.6 ± 26.1	21.2 ± 13.0	16.1 ± 15.4	8.44 ± 15.8
PRI-2201	10	73.1 ± 16.6	72.6 ± 22.8	69.7 ± 18.2 *	67.26 ± 15.6
	1	64.0 ± 23.5 *	40.6 ± 17.3	41.5 ± 15.3 *	36.86 ± 10.5
	0.1	47.8 ± 25.2	28.1 ± 11.3	13.4 ± 14.0	16.50 ± 22.5
PRI-2202	100	36.3 ± 8.7	33.7 ± 8.5	39.2 ± 20.3	32.61 ± 9.2
	10	33.3 ± 39.3	−4.4 ± 16.7	−11.9 ± 28.2	5.78 ± 5.3
	1	36.8 ± 29.3	5.2 ± 26.6	0.8 ± 21.5	5.78 ± 5.3
PRI-2205	100	74.1 ± 16.9	70.7 ± 13.6	65.9 ± 16.5	64.81 ± 19.0
	10	57.9 ± 27.7	25.5 ± 13.7	22.8 ± 11.0	26.69 ± 16.7
	1	35.0 ± 29.1	13.6 ± 10.8	7.8 ± 8.0	26.69 ± 16.7

* *p* ≤ 0.05 as compared to CIS (ANOVA Kruskal-Wallis test); HL-60 cells were pre-exposed to calcitriol or PRI-2191, PRI-2201, PRI-2202, PRI-2205 (100, 10, 1, 0.1 nM) for 24 h and then incubated with CIS for the next 48 h (10, 1, 0.1,0.01 µg/mL). Mean ± SD (standard deviation) is presented.

In the combined treatment with DOX favorable influence on the inhibition of the proliferation was observed in lower doses of the chemotherapeutic agent. Similarly, like in the case of CIS, this effect was the most striking one also in lower doses of vitamin D compounds (Table 3). We also observed the lowering of the DOX IC_50_ value with the increasing doses of calcitriol or its analogs (Table 4), however this effect was not significant like in the case of CIS.

**Table 2:** The antiproliferative activity of cisplatin used after preincubation with calcitriol or its analogs against HL-60 cells.

Concentration of vitamin D analogs[nM]	IC_50_ of cisplatin [µg/mL] combined with:				
	Calcitriol	PRI-2191	PRI-2201	PRI-2202	PRI-2205
100	-	-	-	1.8 ± 0.00	0.01 ± 0.00
10	0.03 ± 1.07	-	0.06 ± 0.08	1.97 ± 0.80	1.55 ± 0.00
1	1.80 ± 0.59	0.72 ± 0.80	1.07 ± 0.75	2.80 ± 1.46	1.79 ± 0.69
0.1	2.85 ± 1.40	2.12 ± 1.02	1.83 ± 0.52	3.56 ± 2.06	2.60 ± 1.46

IC_50_ of cisplatin alone = 4.74 ± 3.22 µg/mL. Mean ± SD (standard deviation) is presented.

**Table 3:** The proliferation inhibition of HL-60 cells exposed to calcitriol or its analogs and doxorubicin.

Compounds	Vit. D analogs [nM]	Concentration of doxorubicin (DOX) [µg/mL]				
		1	0.1	0.01	0.001	0
DOX	0	85.5 ± 1.8	39.9 ± 2.6	23.1 ± 9.4	10.9 ± 3.4	-
calcitriol	10	89.2 ± 14.2	74.4 ± 21.6	65.0 ± 21.6	60.4 ± 41.2	64.9 ± 15.2
	1	88.6 ± 18.8	68.0 ± 18.6	38.6 ± 9.5	26.2 ± 22.0	20.1 ± 7.9
	0.1	89.6 ± 19.9	55.5 ± 33.8	15.4 ± 2.9	6.9 ± 8.5	−4.2 ± 12.4
PRI-2191	10	91.6 ± 12.4	81.6 ± 20.3	74.9 ± 18.6	68.8 ± 35.5	74.1 ± 13.8
	1	91.6 ± 16.1	81.1 ± 19.6	58.6 ± 28.3	67.0 ± 41.1	41.4 ± 6.9
	0.1	89.1 ± 19.2	64.4 ± 37.0	31.5 ± 31.7	39.5 ± 30.1	8.4 ± 15.8
PRI-2201	10	90.4 ± 17.1	75.8 ± 21.0	69.7 ± 22.4	57.0 ± 42.2	67.3 ± 15.6
	1	92.2 ± 19.8	70.0 ± 30.4	67.4 ± 28.4	42.5 ± 33.6	36.9± 10.5
	0.1	90.6 ± 20.8	56.2 ± 37.9	23.8 ± 21.2	4.6 ± 0.4	16.5 ± 22.5
PRI-2202	100	83.9 ± 19.3	57.6 ± 27.0	35.9 ± 6.7	17.8 ± 0.0	32.6 ± 9.2
	10	86.9 ± 20.3	47.1 ± 42.7	14.1 ± 26.0	−12.4 ± 0.0	5.8 ± 5.3
	1	85.9 ± 22.1	44.4 ± 49.7	17.2 ± 26.8	1.3 ± 0.0	8.7 ± 13.5
PRI-2205	100	91.4 ± 13.1	77.0 ± 23.1	70.1 ± 19.5	59.4 ± 31.5	64.8 ± 19.0
	10	91.5 ± 17.6	67.2 ± 31.36	52.7 ± 9.2	43.2 ± 22.2	26.7 ± 16.7
	1	90.7 ± 17.7	2.3 ± 35.1	41.8 ± 25.9	27.1 ± 37.1	14.4 ± 10.9

HL-60 cells were pre-exposed to calcitriol or PRI-2191, PRI-2201, PRI-2202, PRI-2205 (100, 10, 1, 0.1 nM) for 24 h and then incubated with DOX for the next 48 h (10, 1, 0.1, 0.01 or 0.001 µg/mL). Mean ± SD (standard deviation) is presented.

We also analyzed the influence of calcitriol or its analogs used alone or combined with tamoxifen (TX) on the proliferation of human breast cancer cell line MCF-7 (Table 5). Calcitriol, the PRI-2191, PRI-2201 or PRI-2202 used alone inhibit the proliferation of MCF-7 cells in a similar degree. As opposed to HL-60 cells, the analog PRI-2205 was the weakest as the MCF-7 cell proliferation inhibiting agent (Table 5).

**Table 4:** The antiproliferative activity of doxorubicin used after preincubation with calcitriol or its analogs against HL-60 cells.

Concentration of vitamin D analogs[nM]	IC_50_ of doxorubicin [µg/mL] combined with:				
	Calcitriol	PRI-2191	PRI-2201	PRI-2202	PRI-2205
100	-	-	-	0.13 ± 0.09	0.12 ± 0.00
10	0.13 ± 0.00	0.005 ± 0.00	0.08 ± 0.00	0.24 ± 0.18	0.09 ± 0.13
1	0.09 ± 0.05	0.10 ± 0.12	0.15 ± 0.03	0.28 ± 0.25	0.19 ± 0.15
0.1	0.25 ± 0.26	1.91 ± 2.44	1.48 ± 2.20	-	-

IC_50_ of doxorubicin alone = 0.28 ± 0.13 µg/mL. Mean ± SD (standard deviation) is presented.

**Table 5:** The proliferation inhibition of MCF-7 cells exposed to calcitriol or its analogs and tamoxifen.

Compounds	Vitamin D analogs [nM]	Concentration of tamoxifen (TX) [µg/mL]			
		1	0.1	0.01	0	
TX	0	21.6 ± 2.7	5.30 ± 3.3	−2.5 ± 3.3	-
calcitriol	1000				62.9 ± 9.1
	100	62.4 ± 7.4	50.0 ± 10.3 *	47.0 ± 13.3	48.2 ±11.1
	10	32.9 ± 1.0	14.9 ± 3.1	7.2 ± 4.4	6.7 ± 5.4
	1	19.0 ± 4.1	6.7 ± 3.5	-1.7 ± 3.8	−3.3 ± 5.1
PRI-2191	1000				62.4 ± 4.9
	100	60.5 ± 3.6 *	50.4 ± 7.8 *	49.8 ± 9.5 *	48.8 ± 14.2
	10	32.9 ± 1.0	23.6 ± 7.8	17.7 ± 4.8 *	18.1 ± 9.4
	1	19.0 ± 4.1	9.9 ± 2.6	0.6 ± 1.5	2.4 ± 2.4
PRI-2201	1000				58.5 ± 4.9
	100	59.3 ± 4.7 *	50.1 ± 10.3 *	44.6 ± 8.6	44.8 ± 10.9
	10	34.4 ± 2.72	14.9 ± 3.1	10.7 ± 2.9	8.5 ± 5.0
	1	6.7 ± 4.8	6.7 ± 3.5	3.6 ± 3.5	1.6 ± 3.5
PRI-2202	1000				81.0 ± 31.6
	100	69.9 ± 36.4	66.6 ± 41.0	64.1 ± 45.6	64.0 ± 46.1
	10	24.0 ± 6.6	7.8 ± 7.5	1.7 ± 5.5	−0.6 ± 5.2
	1	25.6 ± 7.0	12.0 ± 8.6	2.4 ± 6.1	−0.8 ± 3.1
PRI-2205	1000				64.4 ± 19.6
	100	35.9 ± 6.2	24.5 ± 4.0	17.1 ± 4.8	18.4 ± 8.4
	10	27.0 ± 1.1	9.7 ± 4.0	−2.1 ± 4.4	−1.6 ± 1.1
	1	21.3 ± 2.3	13.4 ± 12.6	−3.0 ± 3.6	−0.96 ± 1.1

* *p* ≤ 0.05 as compared to TX (ANOVA Kruskal-Wallis test). Mean ± SD (standard deviation) is presented.

The test was performed for the range of TX concentrations from 10 to 0.01 µg/mL. At the concentration of 10 µg/mL the proliferation inhibition reached 99% in all treatment protocols used (data not shown). Statistically significant proliferation inhibition as compared to TX alone was observed when calcitriol, PRI-2191 or PRI-2201 were used at 100 nM concentration. Moreover, statistically significant results were also observed when the analog PRI-2191 was used in the concentration of 10 nM (Table 5). Calcitriol, PRI-2191 and PRI-2201 are also able to decrease the IC_50_ value for TX (Table 6).

**Table 6:** The antiproliferative activity of tamoxifen used after preincubation with calcitriol or its analogs against MCF-7 cells (counted as IC_50_).

Concentration of vitamin D analogs [nM]	IC_50_ of tamoxifen [µg/mL] combined with:				
	Calcitrol	PRI-2191	PRI-2201	PRI-2202	PRI-2205
100	0.51 ± 0.00	1.32 ± 1.86	0.21 ± 0.26	-	-
10	1.82 ± 0.01	1.58 ± 0.33	1.76 ± 0.16	2.06 ± 0.34	2.09 ± 0.01
1	2.44 ± 0.16	2.38 ± 0.19	2.12 ± 0.26	2.19 ± 0.36	2.38 ± 0.20

IC_50_ of compounds used alone: TX = 2.34 ± 0.27 µg/mL, cal = 79.11 ± 8.51 nM, PRI-2191 = 90.68 ± 59.28 nM, PRI-2201 = 93.89 ± 0.82 nM, PRI-2202 = 35.19 ± 0.56 nM, PRI-2205 = 408.27 ± 208.61 nM. Mean ± SD (standard deviation) is presented.

To sum up, our recent results have shown that the selected analogs PRI-2205 or PRI-2202 were less potent in antiproliferative activity against HL-60 cells when used alone as compared to the reference compounds. However, among all compounds used, we observed the strongest, but not statistically significant, antiproliferative activity of PRI-2202 and the weakest of PRI-2205 on the human breast cancer cells MCF-7. The ability to potentiate the antiproliferative effect of CIS or DOX against HL-60 or TX against MCF-7 cells was observed in higher doses of the PRI-2202 or the PRI-2205 than those of the reference compounds. Our previous toxicity studies showed that PRI-2202 and PRI-2205 appear to be less toxic analogs than calcitriol and tacalcitol. Even in the total doses of 2.5–5.0 mg/kg (distributed during five successive days), no changes in the body weight were observed. Calcitriol and tacalcitol given in the same protocol showed toxicity at 100-times lower doses. Also, cacipotriol caused the death of all mice (mean life-span ± SD: 7.4 ± 1.1 days) when the total dose of 5.0 mg/kg was administered. Moreover, PRI-2205 revealed no calcemic activity at the doses which inhibit tumor growth or applied at higher doses [18]. Taking into consideration the extremely decreased toxicity of these two analogs, the need for the use of higher doses to obtain similar activity as that of the reference compounds is not critical. 

The mechanism of the antiproliferative activity of calcitriol and its analogs *in vitro* is related to their effect on cell differentiation as well as the deregulation of the intracellular signal transduction and the induction of apoptosis may also be considered [2,28,29,30]. Our previous studies showed that the PRI-2205 appeared to be more active in mouse Lewis lung (LLC) and mammary gland (4T1) tumor growth inhibition than calcitriol, calcipotriol or PRI-2202 [18,31], therefore we decided to analyze the cell cycle and some cell death parameters on the MCF-7 cell line using only the PRI-2205 along with control compounds.

### 2.2. The Effect of Calcitriol or Its Analogs on the Tamoxifen-Induced Cell-Cycle Distribution of MCF-7 Cells

**Figure 3:**
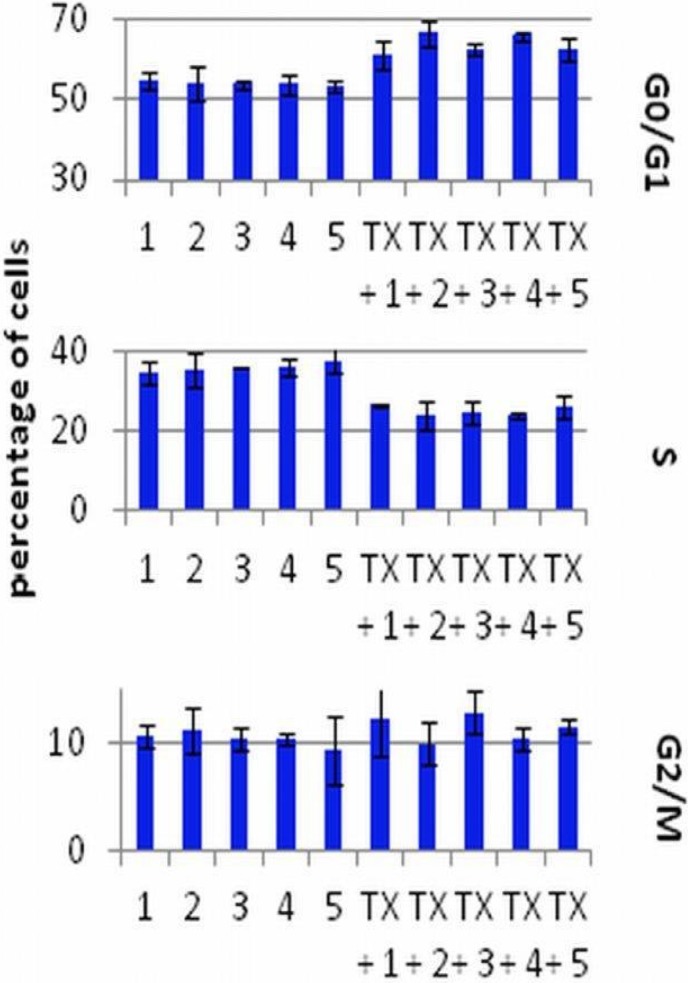
The cell cycle analysis of MCF-7 cells preincubated for 24 h with vitamin D compounds and treated with tamoxifen. TX: tamoxifen (1 µg/mL); 1: control (EtOH); 2: calcitriol; 3: PRI-2191; 4: PRI-2201; 5: PRI-2205. Mean ± SD (standard deviation) is presented. Data were analyzed in WinMDI 2.9 program [32].

The results of DNA analysis in FACS are summarized in Figure 3. The cells were exposed to 1 or 8 µg/mL (data not shown) of TX and 10 nM of calcitriol or its analogs. TX alone in both concentrations used (48 h incubation) increased the number of cells in the G_0_/G_1_ and decreased in the S stage. The 72 h exposition of the MCF-7 cells to calcitriol or its analogs alone did not influence the cell cycle. Only in the lower dose of TX in combination with calcitriol or PRI-2201 caused an increase in the G_0_/G_1_ and a decrease in the G_2_/M stage as compared to TX alone (Figure 3). 

In our previous studies the induction of differentiation by calcitriol, PRI-2191 and PRI-2201 after 120 h of incubation was observed. The cells acumulated in the G_0_/G_1_ stage. PRI-2202 and PRI-2205 appeared to be less potent in the induction of cancer cells differentiation. They caused the apoptosis of HL-60 cells at the dose of 10 nM, but in a higher dose (100 nM) caused cell differentiation. However, in the case of the MCF-7 breast cancer cell line, the PRI-2205, in contrast to all other analogs, increased the accumulation of cells in the G_2_/M stage [18]. 

### 2.3. The Effect of Calcitriol or Its Analogs on Tamoxifen-Induced Apoptosis of MCF-7 Cells

**Figure 4:**
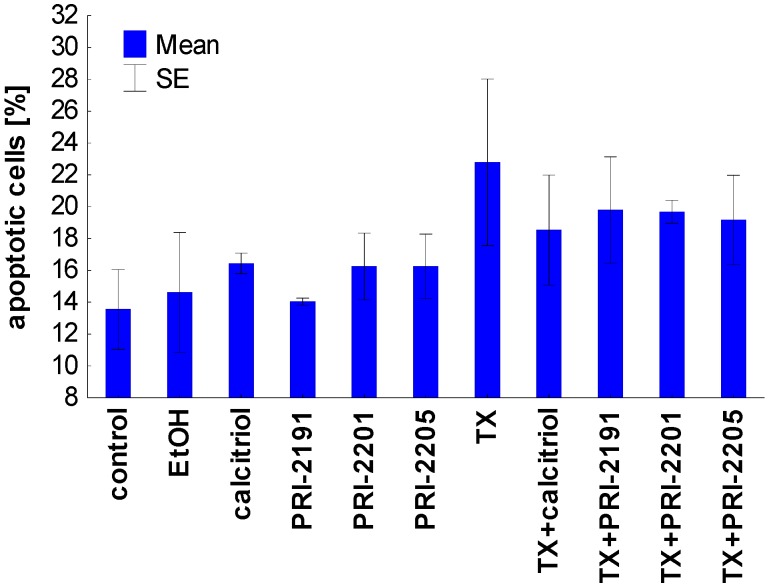
Apoptosis induction by calcitriol or its analogs, alone or used in combination with TX on MCF-7 cells. TX: tamoxifen. The data were displayed as two-color dot plots with FITC-annexin V (FL1-H, Y-axis) *vs.* PI (FL3-H, X-axis)*.* Double-negative cells were live cells, PI^+^/annexin V^−^ necrotic cells, PI weak/annexin V^+^ apoptotic cells, and PI^−^/annexin V^+^ early apoptotic cells. Data were analysed in WinMDI 2.9 program [32]. Figure presents data for PI weak/annexin V^+^ apoptotic cells. The mean and standard error of mean (SE) are presented.

The results of apoptosis analysis after 72 h of incubation are presented in Table 7 and Figure 4. After the analysis of the cells by flow cytometry with the use of annexin V and PI staining, we selected three domains, *i.e*., viable, necrotic and apoptotic cells. The maximal percentage of necrotic cells did not exceed 8% and the changes were similar in all groups (data not shown). Calcitriol analogs were used in concentrations which did not influence apoptosis of MCF-7 cells. However, the combination of TX with calcitriol and its analogs was less potent in inducing apoptosis in MCF-7 cells compared to TX alone (Figure 4). A similar tendency was observed in the mitochondrial potential analysis. The percentage of cells with a high mitochondrial potential decreased with the use of TX, but increased when TX was applied after preincubation with vitamin D analogs. In this analysis the exception was calcitriol, which led to further decreasing the percentage of cells with a high mitochondrial potential (Table 7).

**Table 7:** Mitochondrial membrane potential (Ψmt.) of MCF-7 cells treated with TX alone or combined with vitamin D analogs.

Group	Ψmt.	Group	Ψmt.
Control	80.3 ± 1.8	TX	69.1 ± 11.0
Calcitriol	70.3 ± 2.4	TX + calcitriol	64.9 ± 9.8
PRI-2191	74.4 ± 8.4	TX + PRI-2191	73.8 ± 8.1
PRI-2201	69.6 ± 9.7	TX + PRI-2201	77.0 ± 9.3
PRI-2205	73.2 ± 4.9	TX + PRI-2205	70.1 ± 8.6

Mean ± SD (standard deviation) is presented.

Taken together, the proapoptotic activity of TX, expressed as the diminished mitochondrial membrane potential as well as the increased phosophatidylserine expression, was partially attenuated by vitamin D compounds. These results confirmed the previous studies in which the protective effect of pretreatment with calcitriol or its side-chain-modified analogs on the apoptosis of HL-60 cells induced by cisplatin, doxorubicin, taxol, and genistein was described [33]. It appears that HL-60 cells differentiated by the exposure to calcitriol or its analogs become more resistant to apoptosis [33,34,35,36]. On the other hand, our previous results have also shown that the differentiation of HL-60 cells induced by the pre-exposure to calcitriol or its analogs has not decreased their sensitivity to the antiproliferative effects of doxorubicin, cisplatin, and genistein [37]. In addition, the simultaneous incubation of idarubicin or docetaxel with the PRI-2191 did not decrease the HL-60 or K562 cells’ sensitivity to the antiproliferative activity of these chemotherapeutic agents [38]. In our studies on colon HT-29 cancer cells incubated simultaneously with 5-fluorouracil and vitamin D analogs, a different mechanism was observed in the case of PRI-2205 than with PRI-2191. PRI-2205 combined with 5-fluorouracil significantly increases cell percentage in the S cell cycle phase compared to 5-FU applied alone, whereas PRI-2191 used along with 5-FU caused the accumulation of cells in the G_0_/G_1_ cell cycle phase with a parallel decreasing of cells in the S stage. Moreover, our further studies on MC38/0 mouse colon cancer cells have shown that PRI-2205, but not PRI-2191, indicates a tendency to enhancing cell death induction by 5-fluorouracil, but in parallel both analogs decrease the activity of caspase-3/7 compared to 5-fluorouracil [39]. Simultaneously, both analogs significantly enhanced the antitumor activity of 5-fluorouracil in the MC38 mouse colon cancer model or irinotecan and oxaliplatin in the HT-29 human colon cancer model [39] and [21].

### 2.4. The Influence of Vitamin D Analogs Combined With Chemotherapeutic Agents on the VDR Expression in MCF-7 Breast Cancer Cells *in Vitro*

Since previous studies described a correlation between the VDR expression and proliferation in cultured cells, it was of interest to determine whether the combined treatment schedules used on MCF-7 cells affecting cell growth could reflect the VDR expression. On the other hand, the treatment of T47D cells with estradiol (E2) also increased the VDR expression. But in the case of cells treated simultaneously with E2 and TX, the expression of VDR was comparable with untreated cells [40]*.*

**Figure 5:**
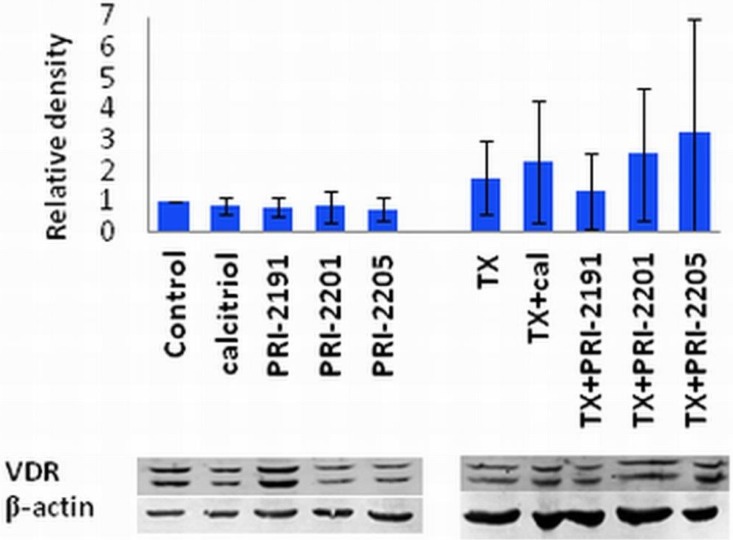
VDR expression in MCF-7 cells treated with TX and vitamin D compounds.The densitometric analysis of the western blots was carried out using ImageJ 1.46r. The blots were normalized to actin and the fold-change protein level expression is reported in comparison to control.

As demonstrated in Figure 5, TX treatment alone causes an increase in the VDR expression, moreover, when calcitriol, PRI-2201 or PRI-2205, but not PRI-2191 where used in combination with TX, we observed further increase in the VDR expression. This observation could partially explain the potentiation of the antiproliferative effect of TX by vitamin D analogs. Our findings correlate with the studies of Abe-Hashimoto *et al*. where it was shown that 22-oxacalcitriol enhances the antitumor activity of TX in the MCF-7 breast cancer model, both *in vitro* and *in vivo* [41]. 

It is documented that approximately 30% of ERα-positive breast cancers do not respond to TX treatment. Moreover, tumors that initially respond to the treatment develop acquired resistance, despite continued expression of ERα [42]. Therefore, combined treatment strategies which sensitize the cells to TX can support breast cancer treatment strategies.

### 2.5. The *In Vivo* Studies

**Figure 6:**
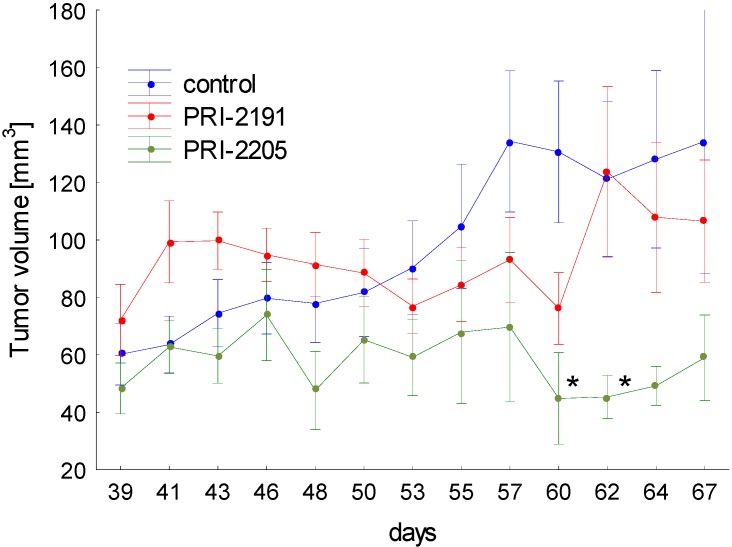
The kinetic of MCF-7 subcutaneous tumor growth. One day before cancer cells inoculation, 90-days release 17-β-estradiol pellets were subcutaneously (s.c.) inoculated. The treatment of NU/J mice bearing subcutaneous human breast MCF-7 tumors were started on day 39 after tumor cells inoculation. PRI-2191 or PRI-2205 were injected s.c. at doses of 1.0 μg/kg/day or 10.0 μg/kg/day, respectively, three times a week, up to day 67 (total dose of PRI-2191: 13 µg/kg; PRI-2205: 130 µg/kg). Means with standard error bars are presented. *p* < 0.05 as compared to control, Mann-Whitney U test.

The results of the tumor growth kinetics were presented in Figure 6. Analog PRI-2205 revealed antitumor activity in mice bearing MCF-7 tumors. Statistically significant inhibition of tumor growth was observed on two days: 60 and 62. Analysis of body weight of mice during treatment showed, that maximal body weight decrease caused by PRI-2205 or PRI-2191 not exceed 4% (data not shown).

These results showing the potency of PRI-2205 to inhibit the growth of transplanted human breast tumors are in accordance with our previous findings from studies on mice tumors (lung LLC and mammary gland 4T1) [18,31]. Moreover we showed, that based only on the *in vitro* results we can eliminate a potential drug candidate.

## 3. Experimental

### 3.1. Analytical Chemistry

The samples of analogs for the analysis were dried in a vacuum drier Salvis Lab VC-20 (Donserv). Nuclear magnetic resonance (^1^H-NMR and ^13^C-NMR) spectra were recorded on Varian GEMINI-200, Varian S 500 and Varian S 600 spectrometers. UV spectra were taken in ethanolic solutions on a Shimadzu UV-160A spectrophotometer. Infrared (IR) spectra were taken on a Perkin-Elmer Model 1725X FT-IR spectrophotometer as films of oily substances or CHCl_3_ solutions. Mass spectra (MS) and high-resolution MS (HRMS) were recorded on a Maldi Spectrometer SYNAPT G2-S HDMS (Waters Corporation, Milford, MA, USA). High-performance liquid chromatography (HPLC) separations were performed using a Knauer Instrument and Eurospher 100 C18 column, 10 µm, 5 cm × 25 cm. Column chromatography was carried out on a Kiesegel 60 (Merck Art. 9025, Darmstadt, Germany).

### 3.2. Synthesis

#### 3.2.1. *(6R/S)-SO_2_-(5E,7E)-(1S,3R)-1,3-bis[t-butyl(dimethylsilyl)oxy]-22-thiobenzothiazolyl-23,24-dinor-9,10-secochola-5(10),7-diene adduct* (**5**)

A solution of the crude alcohol **4** (87 g, 0.14 M) in CH_2_Cl_2_ (500 mL) was prepared and placed in a dropping funnel. A suspension of 2-thiobenzothiazole (45 g, 0.27 M) in CH_2_Cl_2_ (500 mL) was prepared and placed in a cooling bath (0 °C) with a magnetic stirring. Triphenylphosphine (71 g, 0.27 M) was added with stirring in a single portion to this suspension and the solution of the alcohol 4 in CH_2_Cl_2_ was slowly added dropwise. Then diisopropylazadicarboxylate (45 mL, 0.23 M) was added dropwise. The mixture was vigorously stirred for 90 min. at 0 °C. The cooling bath was removed and brine (4 L) and water (200 mL) were added. The organic phase was separated and the residue was extracted with CH_2_Cl_2_ (2 × 200 mL). The combined organic phases were dried over anhydrous Na_2_SO_4_ (80 g). The solution was filtered and concentrated under reduced pressure. The residue was chromatographed on the column with silica gel (230-00 mesh, 800 g, 2%–8% hexane/ethyl acetate). The fractions containing the pure sulphide 5 were concentrated and dried *in vacuo*. The residue (284 g) was dissolved in 2:1 hexane–toluene mixture (600 mL). The suspension was filtered under reduced pressure. The filtrate was concentrated under reduced pressure and thoroughly dried on a vacuum pump. The crude sulphide **5** (100 g, 0.13 M, 93%) was obtained and used for the next step without further purification. ^1^H-NMR (δ, ppm) 0.06 (12H, m, 2Si(CH_3_)_2_), 0.69 (3H, s, 18-CH_3_), 0.88 (18H, m, 2Si-C(CH_3_)_3_), 1.14 (3H, d, *J* = 6.5 Hz, 21-CH_3_), 2.62 and 3.01 (3H, m, 6-H and 22-CH_2_), 3.94 (1H, m, 7-H), 4.19 and 4.37 (2H, m, 1-H and 3-H), 4.69 (2H, m, 10'-CH_2_), 7.34 and 7.77 (4H, m, Ar-H).

#### 3.2.2. *(6RS)-SO_2_-(5E,7E)-(1S,3R)-1,3-bis[Tert-butyl(dimethylsilyl)oxy]-22-sulfonylbenzothiazolyl-23,24-dinor-9,10-secochola-5(10),7-diene adduct* (**6**)

A three neck flask fitted with a mechanical stirrer and a dropping funnel was placed in a water bath. The solution of the crude sulphide **5** (100 g, 0.13 M) in CH_2_Cl_2_ (400 mL) and C_2_H_5_OH (1200 mL) was added. A solution of ammonium heptamolybdenate hydrate (AHT, 22 g, 18.8 mM) in H_2_O_2_ (35%, 150 mL) was dropped in over 5 min. with stirring. The water bath was heated up to 65 °C and the stirring was continued for 2.5 h until the complete disappearance of the substrate (monitored by TLC). The mixture was cooled down to 0 °C and the solution of Na_2_SO_3_ (10%, 900 mL) was dropped in until the disappearance of peroxides (paper indicator). The solvents were removed under reduced pressure, and ethyl acetate (600 mL) was added to the residue. The organic phase was separated and the water phase was extracted twice with ethyl acetate (2 × 300 mL). The combined organic phases were dried over anhydrous Na_2_SO_4_ (40 g) and filtered. The solvents were removed under reduced pressure and the residue was dried on a vacuum pump. The crude sulfone **6** (90 g, 0.11 mol, 85%) was obtained as yellow oil; IR, ν, 2952, 2928, 2883, 2856, 1624, 1472, 1381, 1360, 1323, 1253, 1148, 1121, 1083, 834, 760 cm^−1^; ^1^H-NMR (δ, ppm) 0.06 (12H, m, 2Si(CH_3_)_2_), 0.65 (3H, s, 18-CH_3_), 0.87 (18H, m, 2Si-C(CH_3_)_3_), 1.28 (3H, d, *J* = 6.5 Hz, 21-CH_3_), 3.28 and 3.65 (3H, m, 6-H and 22-CH_2_), 3.94 (1H, m, 7-H), 4.17 and 4.36 (2H, m, 1-H and 3-H), 4.65 (2H, m, 10'-CH_2_), 7.61, 8.02 and 8.22 (4H, m, Ar-H).

#### 3.2.3. (*5E,7E)-(1S,3R)-1,3-bis[Tert-butyl (dimethylosilyl)oxy]-22-sulfonylbenzothiazolyl-23,24-dinor-*9,10-secochola-5,7,10(19)-triene (**7**)

NaHCO_3_ (70 g) was added to the solution of the sulfone **6** (90 g, 0.11 M) in ethanol (1,200 mL) and the mixture was stirred under reflux for 3 h. The mixture was cooled down and the solvents were removed under reduced pressure. Then water (600 mL) and ethyl acetate (600 mL) were added. The organic phase was separated and the residue was extracted with ethyl acetate (2 × 200 mL). The combined organic phases were dried over anhydrous Na_2_SO_4_ (80 g) and filtered. The solvents were removed under reduced pressure. The residue was dissolved in toluene and placed on the chromatographic column with aluminium oxide (1:10) in hexane. Hexane was used to wash out toluene from the column. The product was washed with a 10% mixture of hexane-ethyl acetate. The solvents were removed under reduced pressure to give 42 g of light yellow oily mixture of the crude product **7**. Then ethyl acetate (30 mL) was added and the mixture was heated up near the boiling point and methanol (800 mL) was added in one portion. The mixture was left at −20 °C for 24 h. The precipitate was filtered and washed with cold methanol (90 mL). The precipitate was dried to the constant weight on a vacuum pump. The crude triene **7** (30 g, 0.04 M, 36%) was obtained and used in the next reaction without further purification; UV λ_max_ (EtOH) 271.0, 240.6, 207.0 nm, λ_min_ 245.6, 231.0 nm; IR, ν, 2951, 2928, 2883, 2856, 1636, 1554, 1472, 1324, 1252, 1147, 1083, 834, 760 cm^−1^; ^1^H-NMR (δ, ppm) 0.06 (12H, m, 2Si(CH_3_)_2_), 0.56 (3H, s, 18-CH_3_), 0.85 (18H, m, 2Si-C(CH_3_)_3_), 1.26 (3H, d, *J* = 6.7 Hz, 21-CH_3_), 3.31 and 3.62 (2H, m, 22-CH_2_), 4.22 (1H, m, 3-H), 4.53 (1H, m, 1-H), 4.97 (2H, m, 19E-H and 19Z-H), 5.79 (1H, d, *J* = 11.5 Hz, 7-H), 6.42 (1H, d, *J* = 11.5 Hz, 6-H), 7.62, 8.04, 8.23 (4H, m, Ar-H). 

#### 3.2.4. (*5Z,7E)-(1S,3R)-1,3-bis[Tert-butyl(dimethylsilyl)oxy]-22-sulfonylbenzotiazolyl-23,24-dinor-9,10-secochola-5,7,10(19)-triene* (**8**)

The triene **7** (24 g, 31.8 M) was dissolved in a 5:1 mixture of toluene-methanol (3 L) saturated with argon. Then anthracene (24 g, 134.8 M) was added. The solution was placed in a UV irradiation apparatus and the circulating pump and power supply for the UV lamps were turned on. The irradiation was carried out for 3.5 h at 18–20 °C. The solvents were removed under reduced pressure. Then hexane (400 mL) was added and the residue was left for 6 h at −20 °C. The mixture was filtered *in vacuo*. The residue was dissolved in toluene (450 mL) and the filtered solution of maleic anhydride (2.4 g) in toluene (50 mL) was added. The mixture was saturated with argon and stirred for 12 h on a magnetic stirrer at room temperature. The solvents were removed under reduced pressure. The residue was dissolved in the mixture of toluene (15 mL) and hexane (15 mL) and put on the chromatographic column filled with 350 g of silica gel 230–400 mesh. Mixtures of hexane-ethyl acetate (1%–4%) were used as eluents. The crude product (21 g) was obtained as yellow precipitate. The precipitate was dissolved in ethyl acetate (30 mL) heated up near the boiling point and methanol (700 mL) was added. The solution was left at −20 °C for 24 h. The precipitate was separated using a Buchner funnel and dried to the constant weight on a vacuum pump. The triene **8** (18 g, 23.8 mM, 75%) was obtained as colorless fluffy powder; UV λ_max_ (EtOH) 268.2, 240.0, 214.4 nm, λ_min_ 245.6, 231.0 nm; IR, ν, 2951, 2928, 2883, 2856, 1636, 1554, 1472, 1324, 1252, 1147, 1083, 834, 760 cm^−1^; ^1^H-NMR (δ, ppm) 0.06 (12H, m, 2Si(CH_3_)_2_, 0.55 (3H, s, 18-CH_3_), 0.87 (18H, m, 2Si-C(CH_3_)_3_), 1.26 (3H, d, *J* = 6.6 Hz, 21-CH_3_), 3.28 and 3.65 (2H, m, 22-CH_2_), 4.18 (1H, m, 3-H), 4.36 (1H, m, 1-H), 4.83 (1H, m, 19*Z*-H), 5.16 (1H, m, 19*E*-H), 5.99 (1H, d, *J* = 11.4 Hz, 7-H), 6.21 (1H, d, *J* = 11.4 Hz, 6-H), 7.61, 8.02, 8.22 (4H, m, Ar-H).

#### 3.2.5. *(5Z,7E,22E)-(1S,3R,24R)-24-Cyclopropyl-9,10-secochola-5,7,10(19),22-tetraen-1,3,24-triol* (*PRI-2202,* **2**)

The triene 8 (4.0 g, 5.3 M) was dissolved in 1,2-dimethoxyethane (DME, 40 mL). The flask was placed in a cooling bath (−68 °C) on a magnetic stirrer under argon. A solution of lithium bis(trimethylsilyl)amide (1.0 M in THF, 5.6 mL, 5.6 M)) was added drop-wise with a syringe with stirring. The stirring was continued for 30 min. at −68 °C. Then the (*R*)-2-(*tert*-butyldiphenylsilyloxy)-2-cyclopropylacetaldehyde **9** (2.0 g, 5.9 M) was slowly added dropwise. The cooling bath was removed after 30 min. and the reaction mixture was stirred for additional 24 h at ambient temp. Brine (20 mL) was added, the organic phase was separated and dried over Na_2_SO_4_ (10 g). The solvents were removed under reduced pressure. The crude intermediate **10** (4.1 g, 5.6 mM) was used for the next step without further purification. The intermediate **10** (4.1 g, 5.6 mM) was dissolved in THF (40 mL) under argon. The solution was warmed up to 60 °C with stirring. Tetrabutylammonium fluoride solution (1 M in THF, 15.7 mL, 15.7 M) was added dropwise and the stirring was continued for 2.0 h. The solution was cooled down to 20 °C and 20 mL of brine was added. The organic phase was separated, dried over Na_2_SO_4_ (10 g), filtered and condensed under vacuum. The residue was filtered through silica gel (50 g). The solvents were removed under vacuum and the resulting solid was crystallized from ethyl acetate (30 mL). The triol **2** was obtained (850 mg, 2.1 mM, 40% from 8), as colorless crystals of 99.5% purity (HPLC); UV λ_max_ (EtOH) 264.6, 213.4 nm, λ_min_ 228.6 nm; IR ν 3401, 2949, 2927, 2869, 1631, 1432, 1376, 1325, 1246, 1156, 1117, 1064, 981, 911, 797 cm^−1^; ^1^H-NMR (500 MHz, CDC1_3_) δ ppm: 0.23, 0.32, 0.51 (4H, m, 2'-H and 3'-H ), 0.57 (3H, s, 18-CH_3_), 0.98 (1H, m, 1'-H), 1,04 (3H, d, *J* = 6.9 Hz), 3.47 (1H, dd: 6.9, 6.8, 24-H), 4.23 (1H, m, 3-H), 4.43(1H, m, 1-OH), 5.00 (1H, bs, 19*Z*-H), 5.33 (1H, bs, 19*E*-H), 5.46 (1H, dd, *J* = 6.0, 14.5 Hz, 23-H), 5.54 (1H, dd, *J* = 8.0, 15.5 Hz, 22-H), 6.01 (1H, d, *J* = 11.3 Hz, 7-H), 6.37 (1H, d, *J* = 11.3 Hz, 6-H); ^13^C-NMR (500 MHz, CDCl_3_) δ: 1.82, 2.98, 12.27, 14.19, 17.53, 20.53, 22.23, 23.55, 27.63, 29.06, 39.78, 40.36, 42.89, 45.29, 45.91, 56.19, 56.33, 66.86, 70.83, 111.76, 117.12, 124.94, 128.92, 132.98, 137.77, 142.95, 147.66; HRMS (EI): calcd for C_27_H_40_0_3_ [M^+^]: calc. 412.2977, found 412.2979. 

#### 3.2.6. *(5E,7E,22E)-(1S,3R,24S)-24-Cyclopropyl-9,10-secochola-5,7,10(19),22-tetraen-1,3,24-triol* (*PRI-2205*, **3**)

A solution of LiHMDS (1.0 M in THF, 2.5 mL, 2.5 mM)) was added dropwise using a syringe with stirring to the solution of the triene **7** (1.8 g, 2.4 mM) in THF (10 mL) at −68 °C under argon. The stirring was continued for 30 min. (*S*)-2-(*tert*-Butyldiphenylsilyloxy)-2-cyclopropylacetaldehyde **11** (0.9 g, 2.9 mM) was slowly added dropwise. The cooling bath was removed after 30 min. and the reaction mixture was stirred for additional 24 h at ambient temperature. Brine (5 mL) was added, the organic phase was separated and dried over Na_2_SO_4_ (8 g). The solvents were removed under reduced pressure. The crude intermediate **12** (2.0 g, 2.3 mM) was used for the next step without further purification. The intermediate **12** (2.0 g, 2.3 mM) was dissolved in THF (10 mL) under argon. The solution was warmed up to 60 °C with stiring. A solution of tetrabutylammonium fluoride (1 M in THF, 7.0 mL, 7.0 mM) was added dropwise and the stirring was continued for 2.0 h. The solution was cooled down to 20 °C and 5 mL of brine was added. The organic phase was separated, dried over Na_2_SO_4_ (5 g), filtered and condensed under vacuum. The residue was filtered through silica gel (15 g). The solvents were removed and the resulting solid was crystallized from ethyl acetate (20 mL). The triol **3** was obtained (430 mg, 1.04 mM, 43%) as colorless crystals of 99.2% purity (HPLC); UV λ_max_ (EtOH) 273,4, 209,2 nm, λ_min_ 231,6 nm; IR ν 3570, 3447, 2945, 2871, 1623, 1433, 1373, 1292, 1078, 1227, 1052, 1024, 983, 951, 901, 890, 871 cm^−1^; ^1^H-NMR (600 MHz, CDC1_3_) δ ppm: 0.13, 0.26, 0.40, 0.48 (4H, m, 2'-H and 3'-H ), 0.51 (3H, s, 18-CH_3_), 0.89 (1H, m, 1'-H), 0.98 (3H, d, *J* = 6.6 Hz), 3.34 (1H, dd: 7.8, 7.2, 24-H), 4.09 (1H, m, 3-H), 4.39(1H, m, 1-OH), 4.89 (1H, s, 19*Z*-H), 5.04 (1H, s, 19*E*-H), 5.20, 5.39 (2H, m, 23-H and 22-H), 5.82 (1H, d, *J* = 11.4 Hz, 7-H), 6.49 (1H, d, *J* = 11.3 Hz, 6-H); ^13^C-NMR (600 MHz, CDCl_3_) δ: 1.73, 3.05, 12.22, 15.34, 17.34, 20.39, 22.12, 23.40, 27.58, 28.90, 36.34, 39.87, 40.24, 41.66, 45.78, 55.95, 56.39, 65.31, 70.55, 109.38, 116.02, 122.72, 128.87, 133.30, 137.79, 144.51, 151.79; HRMS calcd for C_27_H_40_0_3_Na [M + Na]^+^: 435.2872, found 435.2875.

### 3.3. Samples of Analogs for Biological Studies

The following vitamin D analogs were used: calcitriol, PRI-2191, PRI-2201, PRI-2202 and PRI-2205. The synthesis of the analogs PRI-2191 (tacalcitol) and PRI-2201 (calcipotriol) was described elsewhere [17,22,43,44,45]. The samples of the compounds were stored in amber ampoules under argon at −20 °C. Prior to usage the compounds were dissolved in absolute ethanol to the concentration of 10^−4 M,^ and subsequently diluted in culture medium to reach the required concentrations (ranging from 1 to 1,000 nM). The chemotherapeutic agents: tamoxifen free base (Sigma, Steinheim, Germany), doxorubicin (Institute of Biotechnology and Antibiotics, Warsaw, Poland), cisplatin (0.5 mg/mL, Ebewe, Unterach, Austria) were used.

### 3.4. Cells

Human HL-60 (leukemia) and MCF-7 (breast cancer) cell lines were obtained from American Type Culture Collection (Rockville, Maryland, MD, USA). Both cell lines are being maintained in the Institute of Immunology and Experimental Therapy (Wroclaw, Poland). 

HL-60 cells were cultured in RPMI 1640 medium (Gibco, Paisley, Scotland, UK) with 2 mM L-glutamine adjusted to contain 1.5 g/L of sodium bicarbonate, 4.5 g/L of glucose, and 1.0 mM of sodium pyruvate, 10% fetal bovine serum (all from Sigma-Aldrich Chemie GmbH, Steinheim, Germany). The MCF-7 cells were cultured in Eagle medium (IIET, Wroclaw, Poland), supplemented with 2 mM L-glutamine and 1.0 mM of sodium pyruvate, 10% fetal bovine serum, 1% MEM non-essential amino acid solution and 0.8 mg/L of insulin (all from Sigma-Aldrich Chemie GmbH). All culture media were supplemented with 100 units/mL of penicillin and 100 µg/mL of streptomycin (both from Polfa Tarchomin S.A., Warsaw, Poland). The cell lines were grown at 37 °C in the 5% CO_2_ humidified atmosphere. 

### 3.5. *In Vitro* Anti-Proliferative Assay

The cells were placed on 96-well flat-bottom plates (Sarstedt, Inc. Newton, NC, USA) at a density of 1 × 10^4^ cells per well, 1–2 h before the addition of the tested compounds. The cells were exposed for 24 h to various concentrations (1, 10, 100 and 1,000 nM) of calcitriol or its analogs and for the next 48 h to various concentrations of chemotherapeutic agents (0.01, 0.1, 1, 10, µg/mL). The MTT (HL-60) or SRB (MCF-7) assay for evaluating the cytostatic effect was performed as described previously [15].

The percentage of the proliferation inhibition was calculated according to the formula:

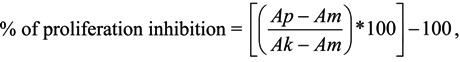
(1)
where:

Ap: the absorbance of treated cells;
Am: the absorbance of culture medium;
Ak: the absorbance of control cells.

The results were also calculated as the IC_50_ (inhibitory concentration 50%), *i.e.*, the dose of the tested compound which inhibits the proliferation of cancer cells by 50%. IC_50_ values were calculated for each experiment separately and the mean values ± *SD* are presented. Each compound at a given concentration was tested in triplicates in a given experiment; each experiment was repeated 3–5 times. Ethanol, which was used as a solvent (in the dilution corresponding to its highest concentration applied to the tested compounds), did not exert any inhibitory effect on the cell proliferation.

### 3.6. Cell Cycle Analysis

The cultured MCF-7 cells were seeded at the density of 1 × 10^5^ cells/mL of culture medium on 24-well plates (Sarstedt, Nümbrecht, Germany) to the final volume of 2 mL. The cells were exposed to the calcitriol or its analogs at the concentration of 10 nM for 24 h. Tamoxifen was added in the concentration 1 or 8 µg/mL for the next 48 h. After 72 h of incubation, the cells were collected (using trypsin/EDTA), washed in phosphate-buffered saline (PBS) and counted in a hemacytometer. The cells (1 × 10^6^) were washed twice in cold PBS and fixed for 24 h in 70% ethanol at −20 °C. Then the cells were washed twice in PBS and incubated with RNAse (8 μg/mL, Fermentas, St. Leon-Rot, Germany) at 37 °C for 1 h. The cells were stained for 30 min. with propidium iodide (0.5 mg/mL, Sigma-Aldrich Chemie GmbH) at 4 °C and the cellular DNA content was determined by flow cytometry using the Cell Quest program (Becton Dickinson, San Jose, CA, USA). Data were analysed in ModFit LT 3.1. 

### 3.7. Apoptosis Determination by Annexin V Staining

The cultured MCF-7 cells were seeded at a density of 1 × 10^5^ cells/mL in culture medium on 24-well plates (Sarstedt) in the final volume of 2 mL. After 1–2 h of incubation, the cells were exposed to calcitriol or its analogs at 10 nM. After 24 h of the incubation 1 µg/mL of tamoxifen was added for the next 48 h. Ethanol itself (the solvent for the compounds tested), in a dilution corresponding to its highest concentration used for the compounds, exerted no toxicity on the cell line studied. After 72 h of the incubation, the cells were collected (using Cell Dissociation Solution Non-enzymatic, Sigma-Aldrich Chemie GmbH), washed in PBS containing 2% FBS and counted in a hemacytometer. The cells (2 × 10^5^) were washed twice with HEPES buffer (10 mM HEPES/NaOH, pH 7.4, 150 mM NaCl, 5 mM KCl, 1 mM MgCl_2_, 1.8 mM CaCl_2_). Next the cells were suspended in 0.2 mL solution of FITC-annexin V (Alexis Biochemicals, San Diego, CA, USA) 100 times diluted in the Hepes buffer (binding buffer) and incubated during 15 min in the dark at room temperature. Prior to the analysis 20 µL of the PI solution (0.1 mg/mL) was added to give the final concentration of 0.01 mg/mL. Data analysis was performed by flow cytometry using the CellQuest program for data acquisition. The data were displayed as two-color dot plots with FITC-annexin V (FL1-H, Y-axis) *vs*. PI (FL3-H, X-axis)*.* Double-negative cells were the live cells, PI^+^/annexin V^−^ necrotic cells, PI weak/annexin V^+^ apoptotic cells, and PI^−^/annexin V^+^ early apoptotic cells. The data were analysed in WinMDI 2.9 program [32].

### 3.8. Mitochondrial Membrane Potential Determination

The MCF-7 cells were seeded at the density of 1 × 10^5^ cells/mL of culture medium on 24-well plates (Sarstedt) to the final volume of 2 mL. The cells were exposed to the vitamin D compounds at concentrations 10 nM during 24 h. After that time, tamoxifen was used at the concentration of 1 µg/mL for next 48 h. Ethanol as a solvent for all compounds, diluted corresponding to its highest concentration applied for the compounds, produced no toxicity. After 72 h of the incubation, the cells were collected (using tripsin/EDTA), washed in phosphate-buffered saline (PBS) and counted in a hemacytometer. Mitochondrial injury was assessed by JC-1 (Sigma-Aldrich) staining. The MCF-7 cells (5 × 10^5^) were washed in phosphate-buffered saline (PBS) containing 2% FBS. The pelleted cells were resuspended in 100 µL of warm cultured medium with the addition of 10 µL JC-1 (the final concentration of JC-1 was 3 µg/mL) and were incubated for 15 min at 37 °C. Next the cells were washed with 1 mL of PBS + 2% FBS and then resuspended in 300 µL of PBS + 2% FBS. The mitochondrial membrane potential was analyzed by flow cytometry using the CellQuest program. The data were analysed in WinMDI 2.9 program. As the positive control of cells with the low potential we used cells which were incubated for 24 h with valinomycin (Sigma-Aldrich) at the concentration of 1 mM.

### 3.9. Western-Blot Analysis of VDR Expression

The MCF-7 cells were exposed to the vitamin D compounds at the concentrations of 10 nM during 24 h. After that time, tamoxifen was used at the concentration of 1 µg/mL for the next 48 h. After 72 h of the incubation, the cells were washed twice in phosphate-buffered saline (PBS). To determine the protein expression by Western blot, the cells were lysed in RIPA buffer (Sigma-Aldrich Chemie GmbH), supplemented with a complete mixture of protease inhibitors (Sigma-Aldrich Chemie GmbH) and then kept on ice for 15 min. Lysates were cleared by micro centrifugation at 8,000 ×*g*, 10 min. 

Protein concentrations were determined using a protein assay (DC Protein Assay, Bio-Rad Laboratories, Hercules, CA, USA). Equal amounts of protein (50 μg) were separated in 10% SDS polyacrylamide gel and transferred to a nitrocellulose membrane (0.45 Micron, NitroBind; GE Water & Process Technologies, Osmonics, Hopkins, MN, USA). Protein loading and transfer efficiency were monitored via 1% Ponceau S-Red staining. The membranes were blocked overnight (4 °C) in 1% blocking reagent (Membrane blocking agent, Amersham, GE Healthcare, Little Chalfont, Buckinghamshire HP7 9NA, UK) in PBS. The following day the membrane was washed three times (3 × 10 min) with 0.1% PBST (PBS/Tween-20) and then incubated for 1 h at room temperature with the primary antibody: rabbit anti-VDR (Santa Cruz Biotechnology Inc., Santa Cruz, CA, USA) or rabbit anti-actin antibody (Sigma-Aldrich, Poznan, Poland). After the incubation, the blot was washed three times with 0.1% PBST and incubated for 1 h with the secondary antibody: anti-rabbit immunoglobulins fluorescein linked (Amersham, GE Healthcare). After the incubation, the blot was washed three times with 0.1% PBST and incubated for 1 h with the third antibody: anti-fluorescein with alkaline phoshatase conjugated (Amersham, GE Healthcare).Then, the membrane was washed three times with 0.1% PBST and incubated for 30 min with ECF substrate (Amersham, GE Healthcare). Fluorescence was detected using a scanner (Typhoon scanner GH Healthcare). The western blots densitometry analysis was carried out using ImageJ 1.46r (National Institutes of Health, Bethesda, MD, USA).

### 3.10. *In Vivo* Activity

Female 6–8 weeks old NU/J mice, weighing 20–25 g, were supplied by the Jackson Laboratory/Anima Lab (USA). Mice were maintained in specific pathogen-free (SPF) conditions. All experiments were performed according to *EU Directive 2010/63/EU for animal experiments* and were approved by the 1st Local Committee for Experiments with the Use of Laboratory Animals, Wroclaw, Poland. 

One day before cancer cells inoculation, 90 days release 17beta-estradiol pellets from IRA (NE-121, 17β-estradiol, 0.18 mg, 90 days, Innovative Research of America, Sarasota, FL, USA) were subcutaneously (s.c.) inoculated. Human breast cancer MCF-7 cells were harvested with the use of 0.05% trypsin/0.02% EDTA, washed once with PBS and re-suspended in Hank’s medium. A single-cell suspension (1 × 10^7^/200 μL per mouse) with cell viability over 90% was inoculated s.c.

The treatment of NU/J mice bearing subcutaneous human breast MCF-7 tumors were started on day 39 after tumor cells inoculation. PRI-2191 or PRI-2205 were injected s.c. at doses of 1.0 μg/kg/day or 10.0 μg/kg/day, respectively, three times a week, up to day 67 of the experiment (total dose of PRI-2191—13 µg/kg; PRI-2205—130 µg/kg). The mice were sacrificed on day 69 after cells inoculation.

The tumors were measured and mice were weighted three times a week. Tumor volume was calculated using the formula (a^2^ × b)/2, where a = shorter tumor diameter in mm and b = longer tumor diameter in mm. Inhibition of tumour growth was calculated from the following formula: TGI [%] (tumour growth inhibition) = (W_T_/W_C_) × 100—100%, where W_T_ is the median tumor volume of treated mice and W_C_—that of untreated control animals. 

### 3.11. Statistical Evaluation

Statistical analysis was performed using STATISTICA version 7.1 (StatSoft, Inc., Tulsa, OK, USA). The ANOVA Kruskal-Wallis test or one-way ANOVA followed by the Fisher LSD were applied. For *in vivo* results Mann-Whitney U test were used. A *p* value < 0.05 was considered significant.

## 4. Conclusions

The biological activity *in vitro* of the diastereomeric analog PRI-2202 is diminished as compared to the parent compounds like calcitriol, PRI-2201 (calcipotriol) or PRI-2191 (tacalcitol). The selected geometric analog PRI-2205 of low toxicity and considerable *in vivo* activity, seems to have a different mode of action in comparison to the reference compounds used in our studies. Further to explore this finding the next generation of vitamin D analogs is to be conceived containing the triene system of unnatural (5*E*,7*E*) geometry and modified A-ring as well as the side chain.
